# Rickettsiales in the WHO European Region: an update from a One Health perspective

**DOI:** 10.1186/s13071-022-05646-4

**Published:** 2023-01-30

**Authors:** Cristoforo Guccione, Claudia Colomba, Chiara Iaria, Antonio Cascio

**Affiliations:** 1 Department of Health Promotion, Mother and Child Care, Internal Medicine and Medical Specialties, University of Palermo, 90127 Palermo, Italy; 2 Pediatric Infectious Disease Unit, ARNAS Civico-Di Cristina-Benfratelli Hospital, 90127 Palermo, Italy; 3 Infectious Disease Unit, ARNAS Civico-Di Cristina-Benfratelli Hospital, 90127 Palermo, Italy; 4 Infectious and Tropical Disease Unit, AOU Policlinico “P. Giaccone”, 90127 Palermo, Italy

**Keywords:** Rickettsiosis, Spotted fever, One Health, *Anaplasma*, *Ehrlichia*

## Abstract

**Background:**

The availability of molecular techniques has significantly increased our understanding of bacteria of the order Rickettsiales, allowing the identification of distinct species in both vector and host arthropods. However, the literature lacks studies that comprehensively summarize the vast amount of knowledge generated on this topic in recent years. The purpose of this study was to conduct a comprehensive analysis of the distribution of Rickettsiales in arthropod vectors, animals and humans in the WHO European Region in order to provide useful information to predict the emergence of certain diseases in specific geographical areas and to formulate hypotheses regarding the possible pathogenetic role of some rickettsial species in the etiology of human pathological conditions.

**Methods:**

A systematic review of the literature in the PubMed and EMBASE databases was conducted following the PRISMA methodology using the search terms “Spotted fever” OR “rickettsiosis” OR “ricketts*” AND all the countries of the WHO European Region, from 1 January 2013 to 12 February 2022. Only studies that identified rickettsiae in human, animal or arthropod samples using molecular techniques were included in the review.

**Results:**

A total of 467 articles considering 61 different species of Rickettsiales with confirmed or suspected human pathogenicity were analyzed in the review. More than 566 identifications of Rickettsiales DNA in human samples were described, of which 89 cases were assessed as importation cases. A total of 55 species of ticks, 17 species of fleas, 10 species of mite and four species of lice were found infected. Twenty-three species of Rickettsiales were detected in wild and domestic animal samples.

**Conclusion:**

The routine use of molecular methods to search for Rickettsiales DNA in questing ticks and other blood-sucking arthropods that commonly bite humans should be encouraged. Molecular methods specific for Rickettsiales should be used routinely in the diagnostics of fever of unknown origin and in all cases of human diseases secondary to an arthropod bite or animal contact.

**Graphical Abstract:**

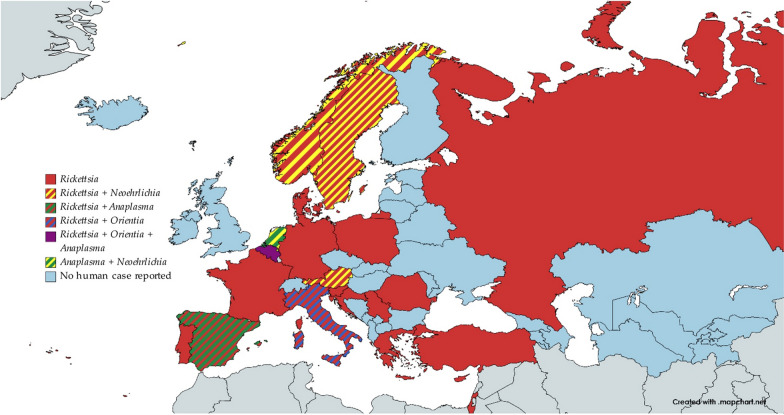

**Supplementary Information:**

The online version contains supplementary material available at 10.1186/s13071-022-05646-4.

## Background

Rickettsioses are vector-borne-diseases caused by microorganisms belonging to the order Rickettsiales. The order Rickettsiales contains a vast number of symbionts of arthropods of unknown or no pathogenicity, as well as species of documented human pathogenic interest, with the latter belonging to the genera *Anaplasma, Ehrlichia, Rickettsia*, *Neoehrlichia* and *Orientia* [1]. The availability of molecular techniques has considerably increased current knowledge on these intracellular pathogens, allowing the identification of different species in both the arthropod vector and the host animal. However, the literature lacks studies that comprehensively summarize the vast amount of knowledge generated on this topic in recent years. The most recent European Centre for Disease Prevention and Control (ECDC) report on the epidemiological situation of rickettsioses in European Union/European Free Trade Association (EU/EFTA) countries, published in 2013, contains no data on arthropod vectors and host animals [2].

The purpose of this study was to conduct a comprehensive analysis of the distribution of members of the order Rickettsiales in arthropod vectors, animals and humans in the WHO European Region in order to provide information that can be used to predict the emergence of certain diseases in certain geographical areas or to formulate hypotheses regarding the potential pathogenic role of some rickettsial species in the determinism of human pathological conditions.

## Methods

**Fig. 1:**
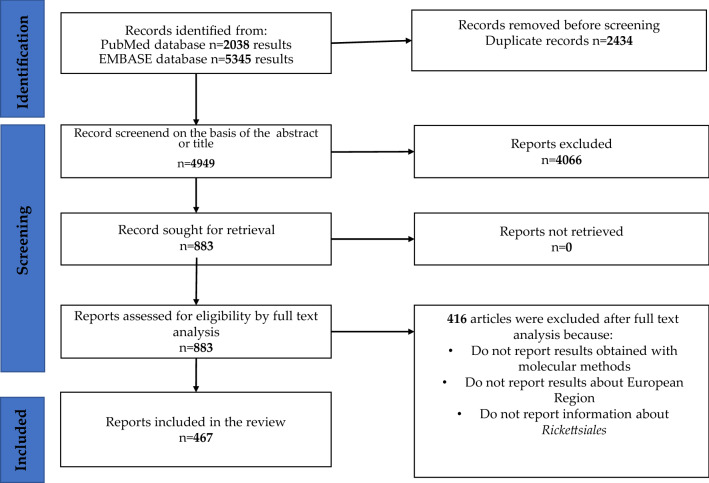
PRISMA flow diagram of article selection for the systematic review [3]

A systematic review of the literature in the PubMed and EMBASE databases was conducted following the PRISMA methodology [3] using the search terms “Spotted fever” OR “rickettsiosis” OR “ricketts*” AND “country” (i.e. a country belonging to the EU; see list in Additional file 1: Table S1). These search terms yielded results describing bacteria belonging to the order Rickettsiales. All WHO European Region countries were included in the search. Reports on extra-European exclaves belonging to a European country (e.g. Mayotte, Falkland Islands, Ceuta and Melilla) were excluded; regions of nations that cross the physical borders of Europe without discontinuity with the remainder of the nation were included (Siberia, Anatolia). Palestine was also included due to the unique nature of its borders with Israel. The search was conducted with no restrictions for relevant articles published between 2013 and February 2022. Figure 1 depicts the bibliographic research process. All articles included in the analysis are listed in Additional file 1: Table S2 and S3, and only the most pertinent are referenced in the text. Only studies that identified Rickettsiales in human, animal or arthropod samples using molecular techniques were included in the review. Only articles describing known or suspected human pathogenic genera (*Anaplasma, Ehrlichia, Neoehrlichia, Orientia* and *Rickettsia*) were included. Articles describing microorganisms known only as a symbiont of arthropods were excluded. The supplementary file consists of an excel file in whose sheets the detailed data relating to the species belonging to Rickettsiales found on vector arthropods, animals and humans can be found. All data can be filtered, thus giving the reader the opportunity to interact freely.

## Results

**Fig. 2:**
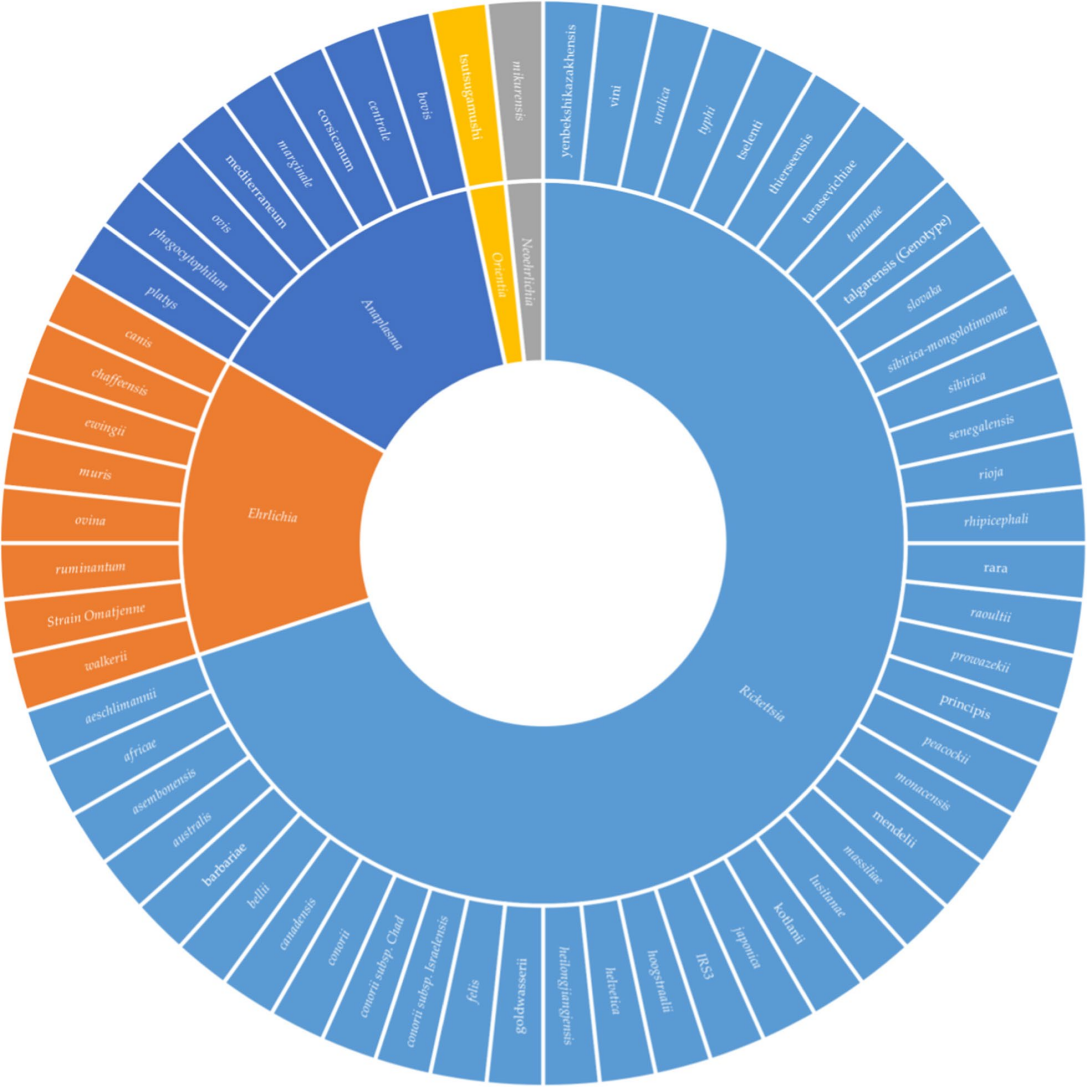
Rickettsiales in arthropod vectors, animals and humans in the WHO European Region. Species lacking a clear taxonomic definition are omitted from the chart and reported analytically in Additional file 1: Table S4, S5 and S6. Species characterized but as yet uncultured (*Candidatus*) are reported in Roman font; other species are shown in italics

A total of 7383 papers were retrieved, of which 883 were examined in full text and 416 were excluded because they did not meet the inclusion criteria. Ultimately, 467 articles were included in the systematic review. Figure 2 depicts 61 distinct species of order Rickettsiales with confirmed or suspected human pathogenicity, of which there were 42 species of *Rickettsia,* eight species of *Ehrlichia* and nine species of *Anaplasma, Neoehrlichia mikurensis* and *Orientia tsutsugamushi. Occidentia massiliensis* was never identified in the WHO European Region. The species found in each country are analytically reported in the Additional file 1: Table S4, S5 and S6.

In this article, we divided the results of our search into the following categories: rickettsiae found in vector arthropods, rickettsiae found in animals, rickettsiae found in human samples.

### Rickettsiae found in arthropod vectors

**Fig. 3:**
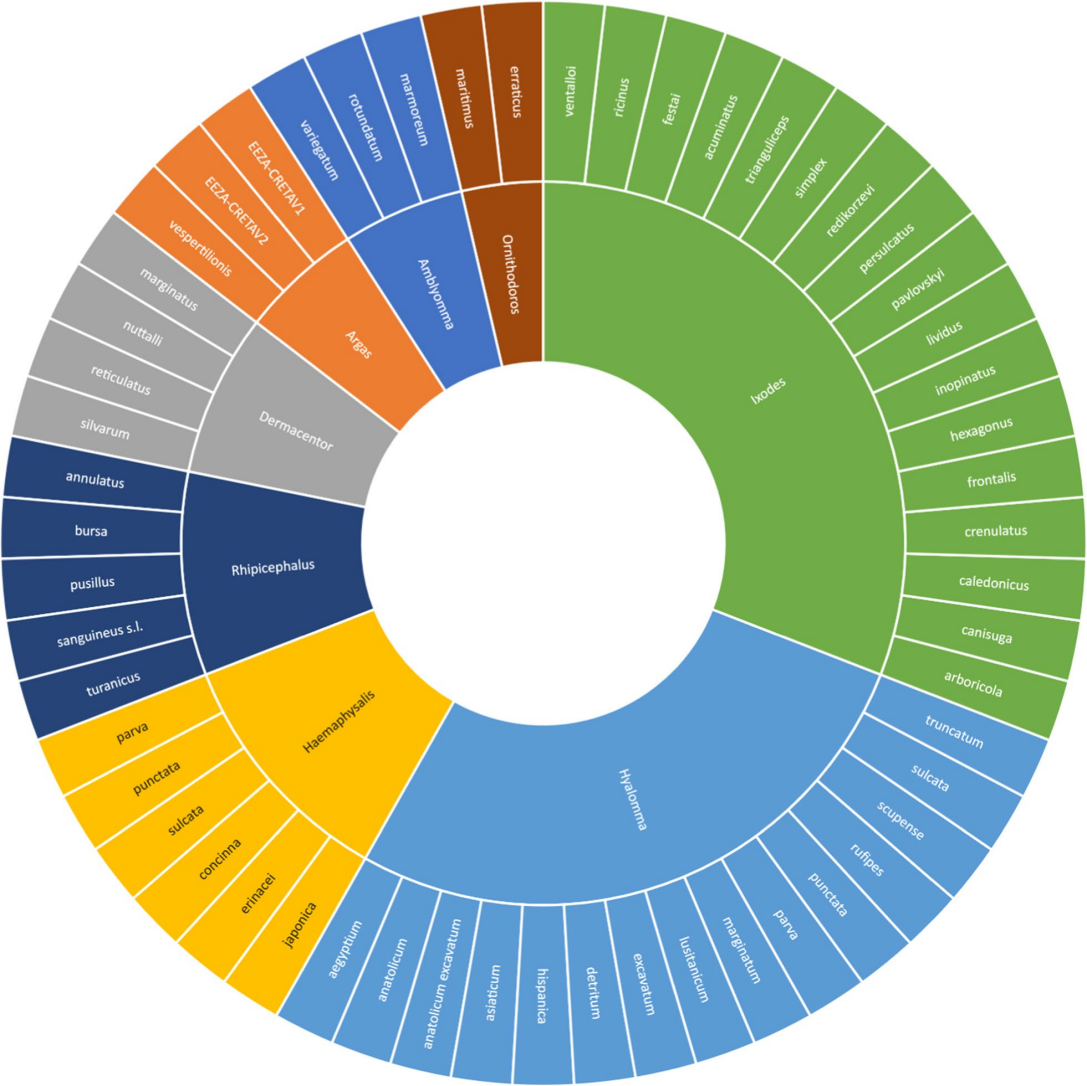
Tick species found to be positive for species of order Rickettsiales

All arthropod species found to be infected with any species of order Rickettsiales are shown analytically in Figs. 3, 5 and 6. In total, 57 species of infected ticks were identified, as well as 17 species of infected fleas, 10 species of infected mites and four species of infected lice. Notably, Rickettsiales bacteria were also discovered in arthropods other than ticks, mites, lice and fleas (conventionally involved in human pathology), including Hippoboscidae *Crataerina pallida* (swift louse fly) [4], *Lipoptena fortisetosa* (deer keds) [5], *Ixodiphagus hookeri* (tick wasp) [6] and *Cephenemyia stimulator* (deer botfly) [7]. Hippoboscidae *Crataerina pallida* was infected with *Rickettsia belli* and *R. monacensis* [4], *L. fortisetosa* with *Rickettsia helvetica* and *Anaplasma phagocytophilum* [5], *I. hookeri* with *Rickettsia helvetica and R. monacensis* [6], and *C. stimulator* with *R. helvetica* [7]. *Ixodiphagus hookeri* is the parasitoid wasp of *Ixodes ricinus*; the positivity of some specimens of these arthropods could be both a result of infection of host ticks as well as a primary infection of the wasp, resulting in the transmission of *Rickettsia* to the ticks and facilitating the spread of microorganisms among arthropods [6]. Future research will be required to determine this wasp's role in Rickettsia ecology.

The identification of rickettsiae was almost always done in hard ticks, with only two genera of soft ticks, *Argas* and *Ornithodoros*, found to be infected and described in few papers. In Europe, various ticks belonging to order Argasidae have been identified and reported [8]. These ticks were *Ornithodoros erraticus* infected with *Rickettsia lusitanae* [9]; *Argas vespertilionis* infected with a *Rickettsia* closely related to *R. africae* [10]; *Argas* EEZA-CRETAV1 and EEZA-CRETAV2 infected with *Rickettsia vini* [8]; *Ornithodoros maritimus* infected with *Rickettsia* sp. [11] identified in Portugal [9], Hungary [10], Spain [8] and France [11], respectively.

**Fig. 4:**
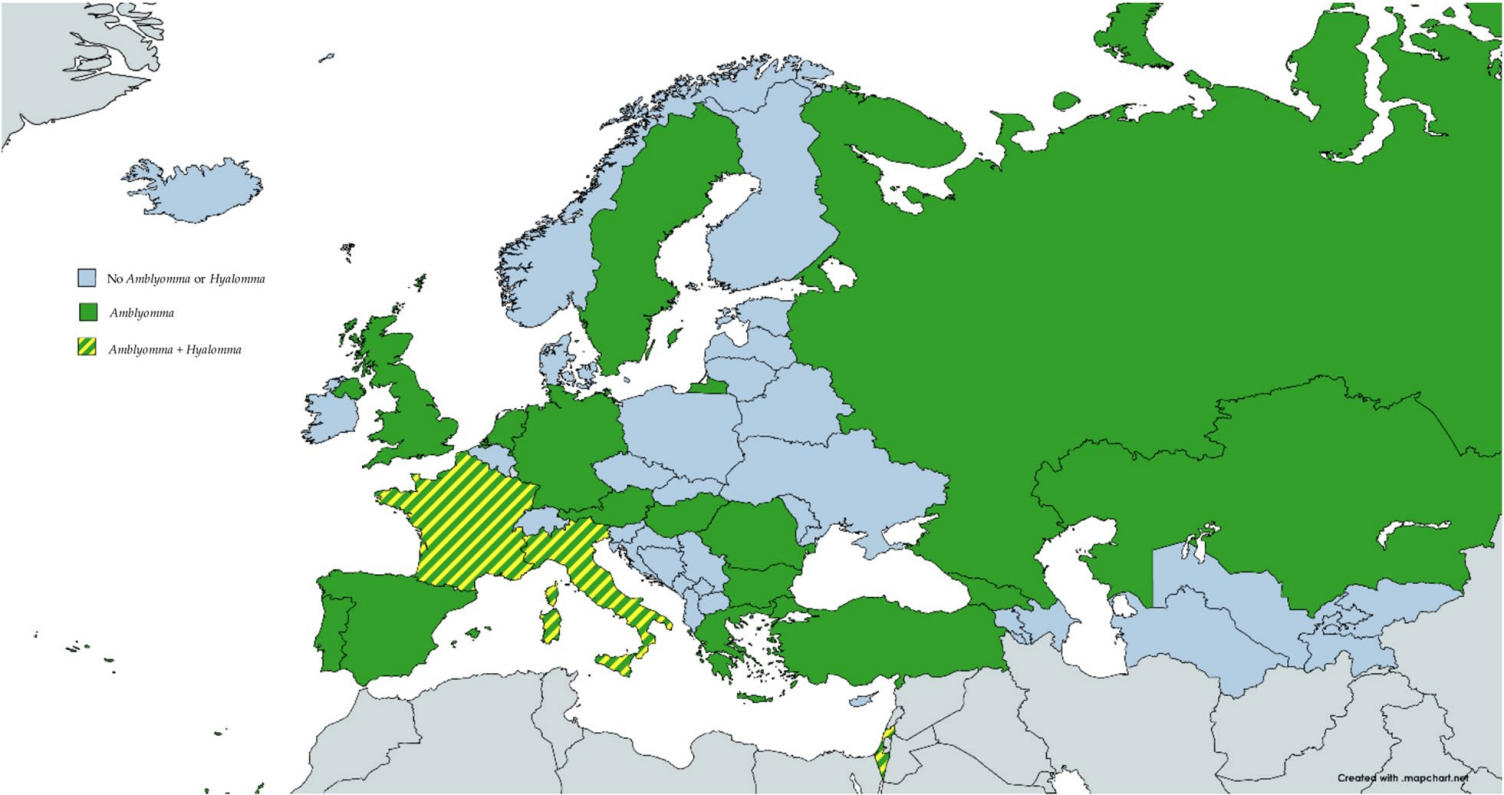
Distribution of *Hyalomma* and *Amblyomma* throughout the WHO European Region. In no country have *Amblyomma* spp. been found in the absence of Hyalo*mma* spp. This chart was made with www.mapchart.net

Also described were ticks belonging to the genera *Amblyomma* [12] and *Hyalomma* [13], which are known as vectors for pathogens that are non-endemic in the majority of European territories. Additional file 1: Table S4, S5 and S6 contain an analytical list of all species and their geographic origin. Species of both *Amblyomma* and *Hyalomma* traveled to Europe via the migration route of birds [14]; *Amblyomma* ticks were identified in southern Europe and Israel and were described feeding on migratory birds at different times; however, these ticks were recently described feeding on cows (in Corsica) [15], sheep (in Sardinia) [12] and turtles (central district of Israel) [16]. Regarding the importation of pathogens, it is notable that *Ehrlichia ewingii* was discovered in a sample of *Hyalomma aegyptium* collected from an illegally imported turtle in southern Italy [17]. Figure 3 summarized the species of ticks described in WHO European Region. Figure 4 provides a graphical summary of the countries in which these two tick genera were found.

Notable is the identification of *Ehrlichia ruminantium* in *I. ricinus* ticks collected from dogs in Russia's North Caucasian Federal district [18]. This is the first and only report of this microorganism in the European region; *Ehrlichia ruminantium* has recently been linked to fatal cases in Africa [19]. *Rickettsia japonica* has only been discovered once in north-eastern Europe [20], but the precise location, animal and tick source of the microorganism's DNA were not specified in this report. *Rickettsia japonica* is the causative agent of Japanese spotted fever (JSF), which is endemic in Japan [21]; in Europe, neither its vector, *Haemaphysalis longicornis,* nor a human case of JSF have ever been described.

**Fig. 5:**
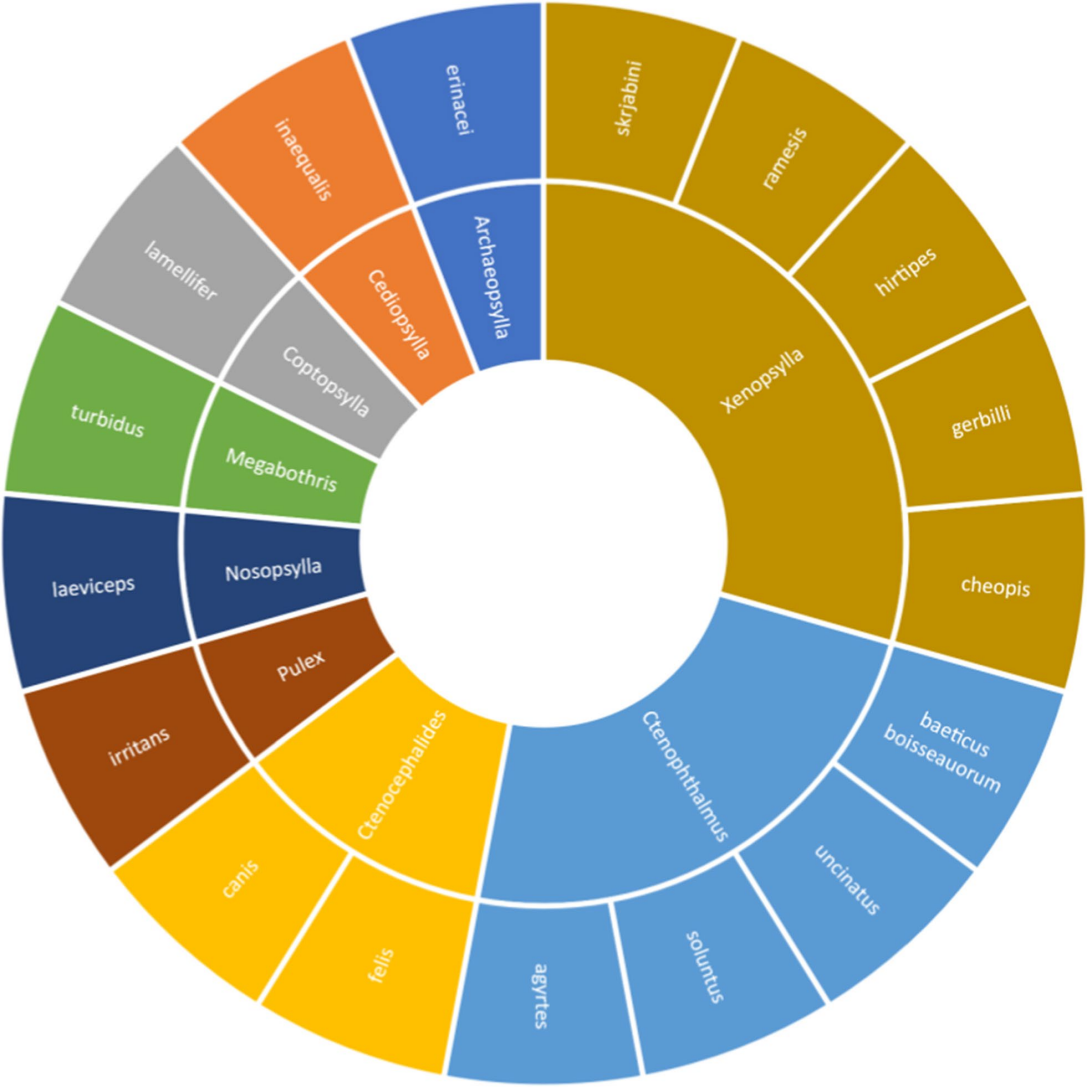
Flea species found to be positive for Rickettsiales

Bacteria of the order Rickettsiales were discovered infecting 12 species belonging to seven genera of fleas. The following 14 bacterial species were identified*: Anaplasma phagocytophilum* [22]*, A. marginale* [23]*, A. ovis* [23]*, Erlichia canis* [23]*, Rickettsia felis* [24]*, R. helvetica* [25]*, R. raoultii* [26]*, R. hoogstraalii* [27]*, R. australis* [27]*, R. asembonensis* [28]*, R. typhi* [24] and three species closely related with *R. felis* [29]*, R. senegalensis* [30] and* Candidatus R. kotlanii* [30]. The host from which the fleas were collected were often pets [31]; less commonly, fleas infected with rickettsiae were collected from hedgehogs [28], rodents [25] and foxes [30]. The flea species in which Rickettsiales DNA was found are shown in Fig. 5.

Only four species of Rickettsiales were identified from mites: *Rickettsia helvetica* [32]*, R. slovaka* [32], *R. monacensis* [33] and *R. felis* [34]. All but one of the identifications were done on mites collected from rodents. This was the report of *Neotrombicula autumnalis* collected in the south of Italy from a lizard and tested positive for *R. monacensis* [33]. This is the sole identification of *R. monacensis* in mites in Europe.

**Fig. 6:**
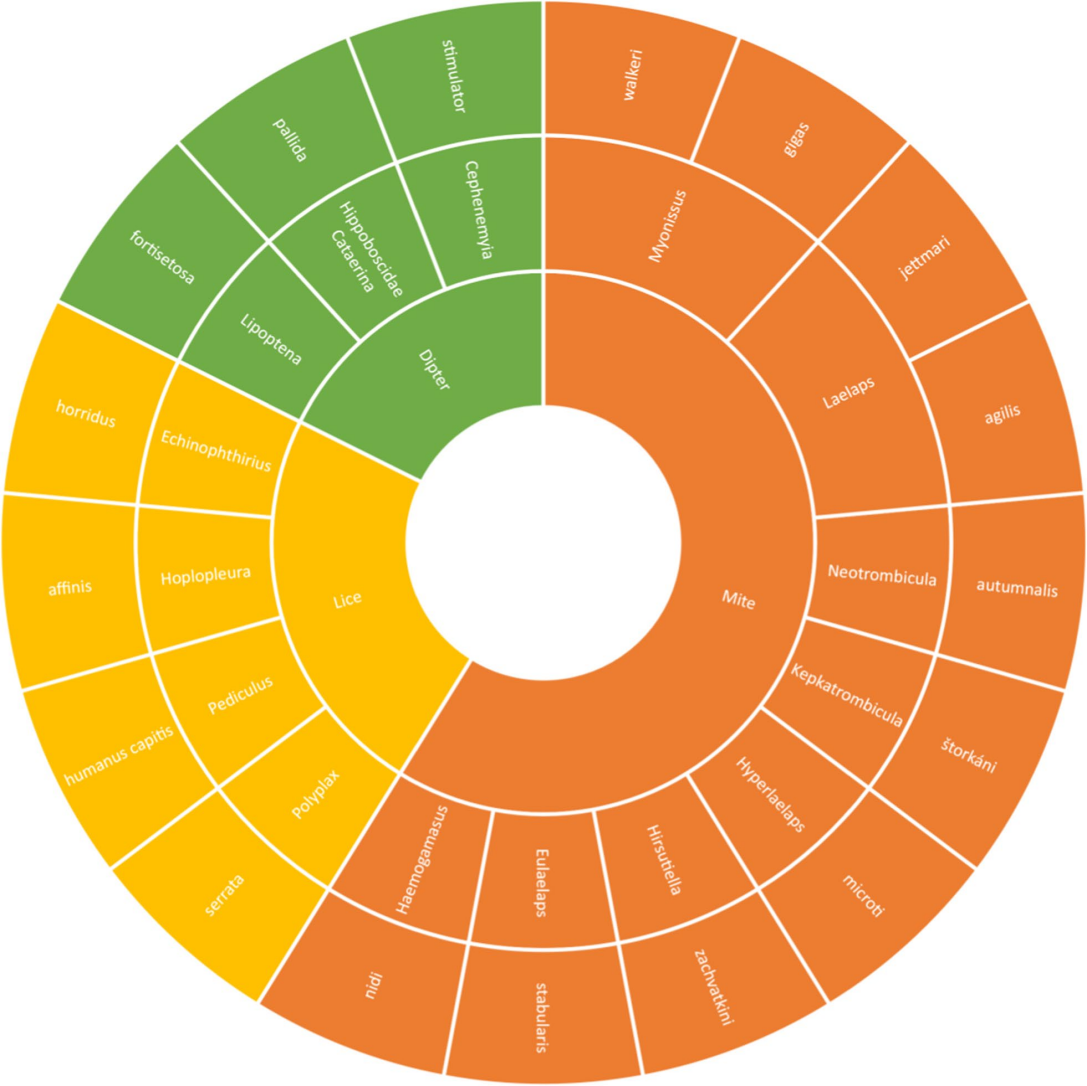
Mite, lice, dipterans and other arthropods found to be positive for Rickettsiales

Regarding lice, *Rickettsia prowazekii* was identified in the louse *Pediculus humanus capitis* in Turkey; this louse was taxonomically identified as *P. h. capitis* by DNA analysis and comparison with the reference strain in the GenBank (AY239286) [35]. The louse *Echinophthirius orridus* was also collected from a seal in The Netherlands, and subsequent study of Rickettsiales DNA showed positivity for *Anaplasma phagocytophilum* [36]. The rodent lice *Polypax serrata* [37] and *Hoplopleura affinis* were found to be infected with *R. helvetica* [37]. Arthropods other than ticks and fleas found to be positive for Rickettsiales DNA are shown in Fig. 6.

### Rickettsiae found in animals

**Fig. 7:**
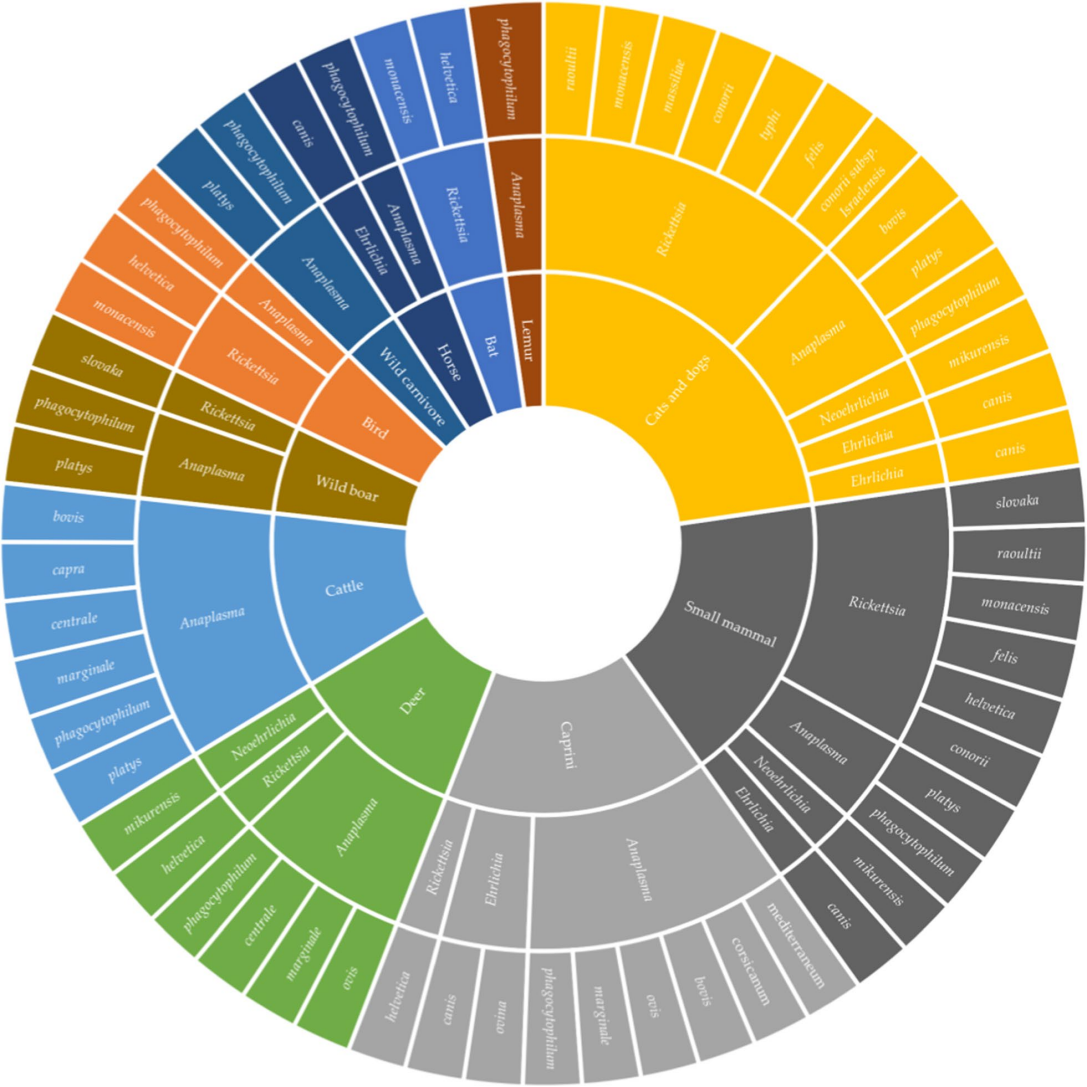
Rickettsiae found in animals. To achieve the greatest visual representation of the chart, cats and dogs are displayed together.* Anaplasma phagocytophilum*, *A. platys*, *Erlichia canis*, *Rickettsia conorii* subsp. *israelensis*, and *Rickettsia typhi* were detected in samples from both dogs and cats, whereas the remaining microorganisms were found in dog samples only. Species characterized but yet uncultured (*Candidatus*) are shown in Roman font; other species are shown in italics

The 23 species of Rickettsiales with clear taxonomic definition that were discovered in samples from wild and domestic animals are shown in Fig. 7. Some species, including *Candidatus A. mediterraneum* and *Candidatus A. corsicanum* have only been discovered in samples from sheep in Corsica [38]; *Ehrilichia* sp. strain Omatjenne was discovered in Turkey in the Black Sea region in samples from asymptomatic and symptomatic cows [39], *Erlichia ovina* was discovered in samples from sheep in central Italy [40] and *Anaplasma capra* in samples from cattle in Kyrgyzstan [41]. In Europe, three species with unclear taxonomy but similarity to known human pathogens have been described: two *Anaplasma phagocytophilum*-like species isolated from clinically ill cows in Turkey [42] and one *Ehrlichia* species closely related to *Ehrlichia chaffeensis* identified in a bird sample in northern Hungary [43]*.* Species of known human pathogenicity identified in animal samples were *Rickettsia typhi* [44]*, R. conorii* [45]*, R. conorii* subsp. *israelensis* [46]*, R. felis* [47]*, R. monacensis* [47]*, Neoehrlichia mikurensis* [48]*, Anaplasma phagocytophilum* [48]*, Rickettsia raoultii* [49]*, R. slovaka* [50] and* R. helvetica* [48]*. Anaplasma phagocytophilum, R. helvetica* and *R. felis* have a wide distribution in Europe among both wild and domesticated animals, as detailed in Additional file 1: Table S6. *Neorickettsia* spp. were found in birds in Hungary and in wild animals in central Europe [43]; Additional file 1: Table S4.

### Rickettsiae found in human samples

In the WHO European Region, over 566 identifications of Rickettsiales DNA from human samples have been documented. In a few of the reports, the exact number of patients was omitted because they belonged to cohorts in which molecular diagnosis was not always performed on all of the subjects. Of a total of 566 patients diagnosed with molecular methods, 405 recovered completely after treatment and 18 died [51]; permanent deficits were reported in two patients [52, 53], and for the remaining patients, either the outcome was unreported or the cohort was so large that the outcome was not described in detail. Negative outcomes were attributed to *Rickettsia conorii* subsp. *israelensis* (12 cases) [51, 54], *R. conorii* (1 case) [55], *R. typhi* (2 cases) [56], a co-infection with *R. sibirica* and *R. tarasevichiae* (1 case) [57], *Candidatus R. tarasevichiae* (1 case) [58] and *R. africae* [59]; the two patients who reported permanent deficit were infected with *Rickettsia sibirica-mongolotimonae* [52] and *R. conorii* subsp. *israelensis* [53]*.* All but one of the 89 cases that were withheld as importation cases originated from outside the European Region [60]. All three identifications of *Orientia* in Europe [60, 61] were found in samples collected from patients who had traveled to Southeast Asia (Cambodia, Vietnam and Laos). Four out of five cases of murine typhus caused by *R. typhi* were reported in travelers returning from Indonesia [61], Cambodia [62], Nepal [62] and Ethiopia [61], while the fifth case was reported in a German traveler returning from Greece [62]. During World War II, *R. typhi* was described in Greece in 12 patients and Germany in two cerebrospinal fluid (CSF) samples from encephalitis-related deaths [56].

In almost 306 cases, diagnostic samples consisted of blood [63]; occasionally, other samples alone or in conjunction with blood have been used, such as eschar swab [64], skin biopsy [65], vesicles swab [66], CSF [67], central nervous system biopsy (autoptic) [56], pleural fluid [68], liver biopsy [62] and in vectors found feeding on patients [69]. On 22 occasions, an infected tick was found while biting the patient’s skin [70]. In four of the 21 instances in which these vectors were tested for Rickettsiales, the same pathogen found in the patient samples was identified [63]; however, in five cases, there was a discordance between the pathogen found in the human sample and that found in the tick [71]. Among 205 patients who underwent serological testing, 134 were positive for *Rickettsia, Rickettsia typhus* group*, Rickettsia conorii, R. helvetica,* and *R. rickettsii* during the acute or convalescent phase. In 69 patients, the results were negative; in 232 cases, the results were not reported, or the assays were not performed; in 131 cases, information was extracted from large cohorts, and it was impossible to determine if the information pertained to patients with a confirmed diagnosis.

Most patients (330) were successfully treated with doxycycline as initial therapy or in combination with other antimicrobials [68]. In 256 patients, tetracyclines were used as monotherapy; in the remaining 72 patients, other drugs were used for empirical treatment to expand the antimicrobial therapy's spectrum [72]. Less commonly used were macrolides, aminoglycosides, β-lactams or chloramphenicol [57].

Therapy was not reported for 204 patients. A full recovery without antimicrobial therapy was described in six cases; these cases were due to *Rickettsia aeschlimannii* (1 case of acute hepatitis from Apulia, Italy) [73], *Neoehrlichia mikurensis* (3 cases in Tyrol, Austria [74] and 2 cases in Sørlandet, Norway [75]. The last two patients were both immunocompromised and affected by autoimmunity; one was infected with *N. mikurensis* and the other with *Rickettsia* sp*.*[75].

Among the patients with a poor outcome, doxycycline was used 5 times; the antimicrobial therapy for intracellular and atypical bacteria was not started promptly due to a delayed diagnosis in two cases [55, 57]. Information about pharmacological treatments was not available in 10 patients who died from Israeli Spotted Fever.

**Fig. 8:**
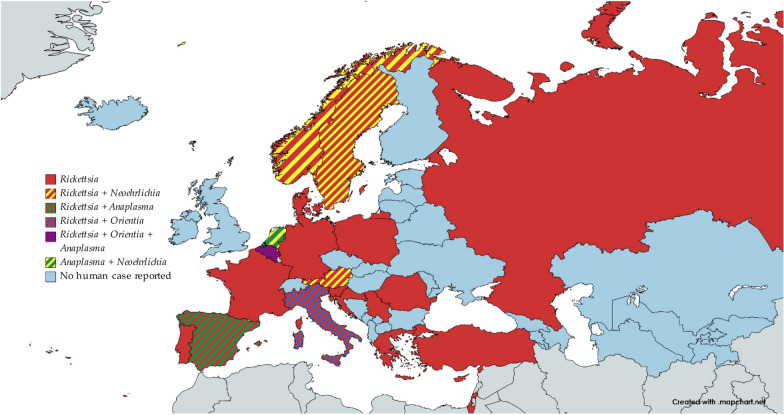
Rickettsiae found in human samples in the WHO European Region. The cases of *Orientia* infections were all imported cases from areas outside the WHO European Region. The chart was made with www.mapchart.net

The countries of the WHO European Region in which cases of rickettsioses, anaplasmosis, ehrlichiosis or scrub typhus were reported are shown in Fig. 8.

## Discussion

The objective of this review was to assess the spread of members of the Rickettsiales in Europe from the One Health perspective [76]. This perspective broadens our understanding of disease through animal surveillance and environmental research, with the goals of early disease detection, a better understanding of threats and risk mitigation.

Historically used as a unique diagnostic tool, the serological assay lacks sensitivity and specificity, particularly in the acute phase of the infection. Molecular methods, although not always highly sensitive, when used on samples taken during the acute phase may lead to the identification of species almost always missed in the past, allowing for a correct linkage between the clinical picture and rickettsial species. Climate change may alter the distribution of the Rickettsiales' animal hosts and arthropod vectors. Migratory birds play an important role in the distribution of rickettsiae. Ticks belonging to *Hyalomma* and *Amblyomma* are not autochthonous to Europe and may carry pathogens that pose a risk to human health, such as *Rickettsia africae, R. sibirica mongolotimonae, R. aeschlimannii* and Crimean-Congo hemorrhagic fever virus [77]. It is well known that these ticks can be found in Europe. While feeding on migratory birds in the summer, these arthropods are transported from sub-Saharan Africa to Europe, and their presence in Europe is anticipated. However, it is also anticipated that these ticks will perish during the winter due to the temperatures encountered, which are not beneficial to their metabolism. It had been hypothesized that due to climatic changes and the marked ability of ticks belonging to the genus *Amblyomma* to colonize new areas, southern Europe would become susceptible to colonization by these ticks, with Sardinia and Sicily identified as areas of particular interest [77, 78]. This prediction has been realized, and ticks belonging to the genus *Amblyomma* have been discovered in Sardinia on animals other than birds and in a stage demonstrating the capacity for reproduction and winter survival [12]. Interest is not limited to the countries bordering the Mediterranean Sea. The presence of *Hyalomma* in Europe has been documented in various EU countries, including those far from the Mediterranean Sea, such as Sweden [79], the UK [80], Germany [81], Austria [82], Romania [83] and Bulgaria [84].

Regarding the spread of the microorganisms, the acute nature of rickettsioses renders human movement less significant than animal movement. Despite this, it is essential to remember that a potentially lethal pathogen could be present in people moving to Europe. Scrub typhus is among the diseases requiring attention. Scrub typhus is a zoonotic disease transmitted by trombiculid mites that is prevalent in South-East Asia [85]. Up to 2013, imported cases of scrub typhus have only been reported in Italy [60] and in Belgium [61], and there are no indications that the disease could become endemic in Europe. Cases of African tick bite fever (ATBF) have been described in European travelers, but autochthonous cases of ATBF have never been described despite the presence of *Rickettsia africae* in ticks, possibly indicating that autochthonous cases will be reported in the coming years. In epidemic typhus, a disease caused by *Rickettsia prowazekii* and whose vector is *Pediculus humanus corporis*, the role of humans as reservoirs is notable [85]. In the past 50 years, no indigenous cases of epidemic typhus have been reported in the WHO European Region, but there are indications that this may soon change. Poor social, economic and hygiene conditions have led to outbreaks of epidemic typhus. Although both *P. humanus capitis* and *P. humanus corporis* were reported in these settings, *P. humanus corporis* was typically the only species analyzed during these outbreaks. However, the ability of *P. humanus capitis* to transmit in vitro *R. prowazekii* has never been described in the field. Some authors theorize that the competence of *R. prowazekii* is lower in the vector *P. humanus capitis* than in *P. humanus corporis* due to the position of the feces, which are more likely to be inhaled from the body than from the head [86]. *Rickettsia prowazekii* has only been identified in Turkey in Europe; its clinical significance is unknown, and additional research on this species is required. Furthermore, *R. prowazekii* could be dormant in adipose tissue for many years and could reactivate under conditions of immune suppression, starvation or intense stress that causes immunity to wane; this condition typically results in a milder illness than the primary infection, known as Brill-Zinsser typhus [87]. The migratory flows from Africa (and from areas where outbreaks of epidemic typhus are described, such as Rwanda, Burundi and Ethiopia) could transport *R. prowazekii* in this manner; consequently, starvation conditions, effects of exhausting travel and the conditions encountered in the refugee camps could reactivate and worsen the already precarious health conditions of these individuals. These data indicate that Europe is not protected from epidemic typhus and that the living conditions of the most socially vulnerable must be addressed. The identification of *R. prowazekii* in Turkey is the only instance in Europe; its clinical significance is unknown, and additional research is required.

**Fig. 9:**
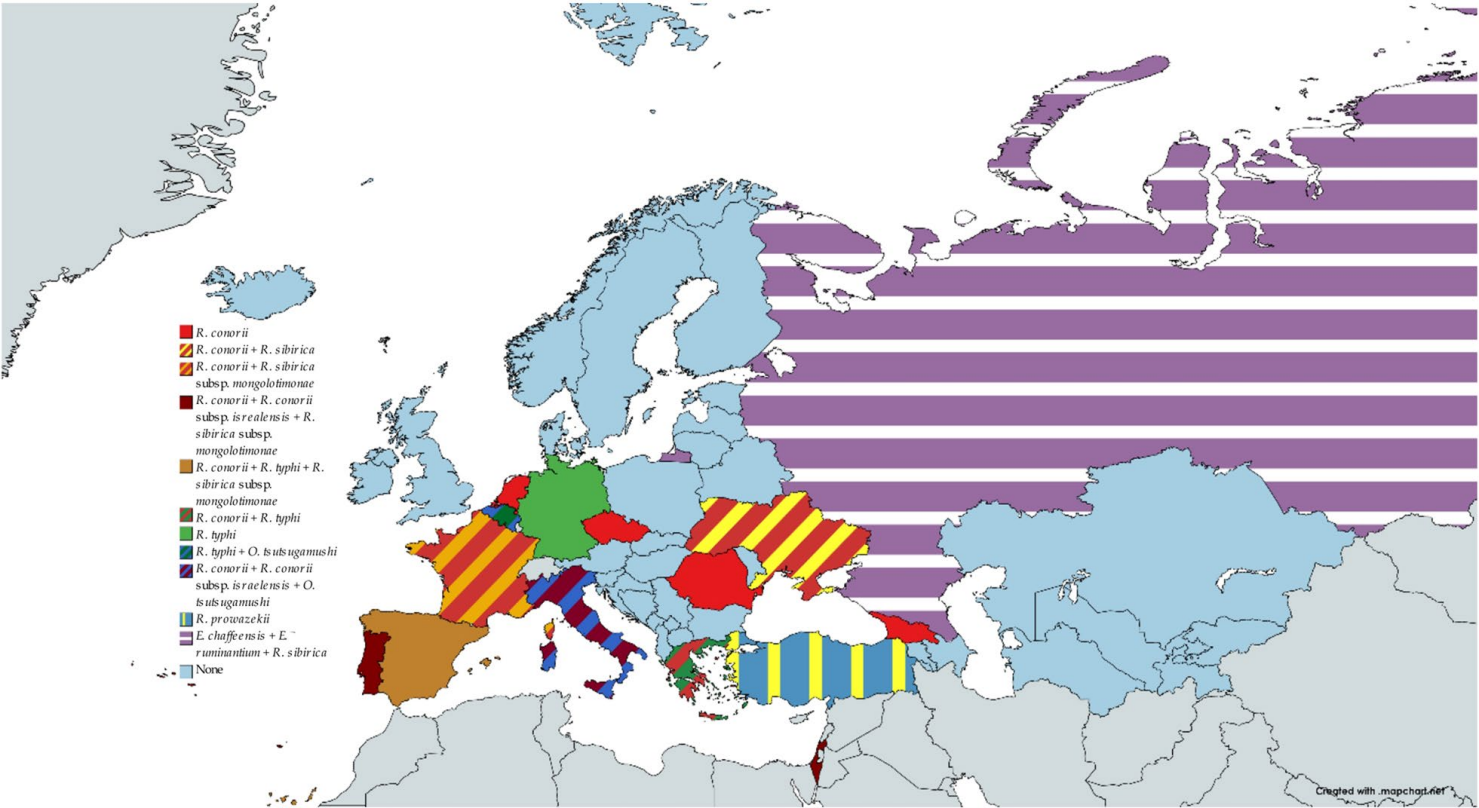
Rickettsiae associated with the most severe human diseases found in human, animal and arthropod samples. Countries are highlighted in their entirety even if some regions within them were not involved. The chart was made with www.mapchart.net

The species described in this article exhibit a wide range of pathogenicity, with some being of unknown pathogenicity, while others can cause both mild and life-threatening diseases. Members of order Rickettsiales linked to the deadliest diseases are *Rickettsia conorii, R. conorii* subsp. *israelensis, R. typhi, R. sibirica, R. sibirica mongolotimonae, R. prowazekii, Erlichia ruminantium* and *E. chaffeensis.* Figure 9 shows the countries in which these species were identified.

The discontinuity between the territory in which these microorganisms were described and neighboring territories suggests that their presence in neighboring countries should be investigated, particularly in the Balkans, which are climatically and geographically suitable for the presence of a wide variety of arthropod vectors but lack specific studies. Countries bordering Asia and Africa, such as Kazakhstan or Azerbaijan, may be more affected by the alterations in wildlife balance and vector ecology than western Europe.

The absence of articles reporting the presence of certain known pathogens in our review may be attributable to the low number of publications in regions where these pathogens are prevalent. The etiological agent of Astrakhan spotted fever (ASF) is *Rickettsia conorii* subsp. *caspia*. This microorganism and cases of African swine fever were described in the Astrakhan region, where it remains endemic (around the northwestern coast of the Caspian Sea, along the border between southern Russia and Kazakhstan), as well as in far-flung nations such as France and Zambia [88]. The first outbreak of ASF in Astrakhan was attributed to industrialization and changes in the ecology of the tick *Rickettsia pusillus* [89]. There was no mention of *R. conorii* subsp. *caspia* in the articles cited in this study.

Rickettsial diseases may be severe and life-threatening. A high index of suspicion is required to initiate effective treatment as soon as possible. Clinical manifestations are not always highly indicative, and the correct diagnosis may be delayed or missed.

Notably, the vector's behavior can affect the disease's characteristics, as in the case with TIBOLA (tick-borne lymphadenopathy), in which the eschar is on the scalp, and ATBF, in which there are multiple eschars [90, 91]. In many rickettsial diseases, the absence of eschar may be due to the involvement of a vector other than ticks. Many arthropods have been found positive for Rickettsiales in Europe, and while the principal vectors of many rickettsial pathogens are known, the role of other arthropods as vectors should be investigated. In this context, the identification of *R. felis* in *Anopheles* mosquitoes in Africa [92] is noteworthy. *Rickettsia felis* was identified in many patients co-infected with *Plasmodium* in western Africa; in that region, the prevalence of *R. felis* infection among febrile patients was reported to range from 3% to 15%, making *R. felis* a potential etiological agent of fever of unknown origin (FUO) [93]. In Europe, *R. felis* infects cats, and numerous cases of infected *Ctenocephalides felis* have been reported; therefore, this microorganism may be the cause of FUO. The possibility of cryptogenic transmission of Rickettsiales through mites, fleas or mosquitoes should be investigated.

The absence of data on the incidence of rickettsial-related diseases may be a limitation of our study, but this was not within the scope of the investigation. In some cases, it was also difficult to accurately assign species of Rickettsiales to the different countries due to the sometimes incomplete descriptions of geographic data provided in the original papers.

## Conclusion

Diverse taxa are responding ecologically and evolutionarily to the accelerating rate of climate change. Knowledge of pathogens, their vectors and vector diffusion is essential for controlling and anticipating the emergence of new diseases in the WHO European Region. To better define the role of members of the order Rickettsiales in human disease, it is crucial to encourage the routine use of molecular techniques to search for these microorganisms in bloodsucking arthropods that commonly bite humans, such as ticks. In addition, the search for Rickettsiales using molecular techniques should be utilized routinely in the diagnosis of FUO and all human diseases caused by arthropod bites. Rickettsiae of unknown or uncertain pathogenicity may one day be associated with distinct clinical manifestations.

## Supplementary information

**Additional file 1.** Key of search.

**Additional file 2.** List of references.

**Additional file 3.** List of references by country.

**Additional file 4.** Rickettsiales found in arthropod vectors.

**Additional file 5.** Rickettsiales found in humans.

**Additional file 6.** Rickettsiales found in animals.

## Data Availability

Not applicable.
